# Dropping risk stratification with subsequent treatment-risk paradox in non ST elevation acute coronary syndromes: a clinical audit in Iraq

**DOI:** 10.1186/s12913-021-07034-7

**Published:** 2021-09-26

**Authors:** Zainab Atiyah Dakhil, Hasan Ali Farhan

**Affiliations:** 1 Department of Medicine, Al Kindy College of Medicine, University of Baghdad, Baghdad, Iraq; 2 Iraqi Scentific Council of Cardiology/ Iraqi Board for Medical Specializations, Baghdad, Iraq; 3 Baghdad Heart Centre/ Medical City, Baghdad, Iraq

**Keywords:** Audit, Guideline adherence, Healthcare policy, Middle East, Risk scores

## Abstract

**Background:**

Risk stratification is the cornerstone in managing patients with Non-ST Elevation Acute Coronary Syndromes (NSTE-ACS) and can attenuate the unjustified variability in treatment and guide the intervention decision notwithstanding its impact on better healthcare resources use. This study sought to disclose real adherence to guidelines in risk stratification of NSTE-ACS patients and in adopting intervention decision in practice.

**Methods:**

Multicentre prospective study recruited NSTE-ACS patients. Baseline characteristics were collected, TIMI (Thrombolysis in Myocardial Infarction) and GRACE (Global Registry of Acute Coronary Events) scores were calculated, management strategy as well as timing to intervention were recorded.

**Results:**

*n.* = 150, 72% of them were males, mean age was (59 ± 12.32) years. TIMI score was calculated in 5.3% of patients with none of them had GRACE score calculated. Invasive strategy was adopted in 85.24 and 82.7% of low GRACE and TIMI risk categories respectively, while invasive approach used in 42.85 and 40% of high GRACE and TIMI risk categories respectively. The immediate intervention in less than 2 hours was more to be used in low-risk categories while the high-risk and very high-risk patients whom were managed invasively were catheterized within >72 h; or more frequently to be non-catheterized at all. Sixty percent of those with acute heart failure, 80.76% of those with ongoing chest pain, 85% of those with dynamic ST changes same as 80% of those with cardiogenic shock were treated conservatively. Using multivariable analysis older age, ongoing chest pain and cardiogenic shock predicted conservative approach.

**Conclusions:**

There is striking underuse of risk scores in practice that can contribute to treatment-risk paradox in managing NSTE-ACS in form of depriving those with higher risk from invasive strategy despite being the most beneficiaries. The paradox did not only involve the very high-risk patients but also the very high-risk criteria like ongoing chest pain and cardiogenic shock predicted conservative approach, this highlights that the entire approach to patients with NSTE-ACS should be reconsidered, regardless of the use of risk scores in clinical practice. Audit programs activation in middle eastern countries can inform policymakers to put a limit to the treatment-risk paradox for better cardiovascular care and outcomes.

## Introduction

According to WHO survey (2017) death from coronary artery disease (CAD) in Iraq reached about 18.5% of total deaths [1]. This big killer; CAD; mainly presented as either chronic stable angina or acute coronary syndrome (ACS). Despite Non ST elevation myocardial infarction (NSTEMI) incidence continues to rise with greater improvement in outcomes of ACS patients in last decades, long term outcomes have not improved in NSTEMI at the same rate of ST elevation myocardial infarction (STEMI) a suggested explanation is depriving those with most benefit from intervention [2]. This necessitates using objective risk stratification tools to guide management plan in NSTE-ACS with TIMI and GRACE scores being most reliable tools, proper risk assessment of NSTE-ACS patients is mandatory to guide the triage among alternative levels of hospital care (e.g., Coronary Care Unit [CCU] vs hospital ward vs outpatient care) [2, 3], as well as decision and timing of intervention [3–6] as following:

*Very high-risk criteria patients (Shock, acute heart failure, ongoing chest pain, life threatening arrhythmia, mechanical complications, or recurrent dynamic Electrocardiogram [ECG] changes) should be catheterised within 2 hours.
*High risk patients (NSTEMI, dynamic ECG changes, GRACE score more than 140) should be catheterised within 24 h.
*Intermediate risk category (diabetes, renal insufficiency, or left ventricular ejection fraction less than 40, post MI angina or prior CABG or GRACE score 109–140) should be catheterised within 72 h.

It is noted that patients who can benefit mostly from treatment are not being treated because there is an exaggerated fear from treatment complications. This phenomenon, often referred to as the ‘treatment-risk paradox’ [7, 8]. Underuse of risk scores may explain this paradox, as health professionals disagree on the importance of cardiac risk scores used to decide on the management of NSTE-ACS patients [9, 10].

This study sought to investigate the adherence of cardiologists to international guidelines in managing NSTE-ACS patients in form of using risk stratification scores (TIMI and GRACE scores) in daily practice in Iraq and to determine if there is treatment-risk paradox in catheterising this population and the predictors of this paradox if present.

## Patients and methods

### Design

Multicentre and prospective study, it is part of clinical audit that was conducted to evaluate all aspects of management of patients with NSTE-ACS.

### Setting and duration

The study conducted in Iraq and included three cardiac centres, that are teaching centres and percutaneous coronary intervention (PCI) capable centres with 24/7-day emergency PCI services. The study started from January 2018 to January 2019. Cases recruitment done sequentially (not simultaneously) during affiliation period of the investigator in each of these three cardiac centres.

### Patient selection

The study *included* patients with confirmed diagnosis of NSTE-ACS (Patients with acute chest pain but no persistent ST-segment elevation) [6], the diagnosis of NSTE-ACS was confirmed by the head of the treating team who was usually a consultant cardiologist.

#### Exclusion Criteria

1. Persistent ST elevation on ECG 2. New or presumed new left bundle branch block (LBBB) 3. Active malignancy 4. End stage renal disease 5. Active Upper GIT bleeding 6. Patients refusing intervention 7. Frail patient 8. Any patient with missed data (regarding in-hospital follow ups and checking for development of the high-risk features, time to catheterization lab) was excluded from the study.

### Role of investigator in cases selection and data collection

The investigator registered the case, TIMI and GRACE risk scores were calculated by the investigator, then the investigator attended the daily morning rounds and follow-up meetings with the treating team and evaluated the management plans and determined whether or not the treating team calculated the risk scores during any step in management. So, researchers did not only depend on patients’ records, to overcome the limitation that was reported by prior researchers [4] in that checking only risk scores from patients’ records would not reflect the real-world practice thus even verbal score calculation during daily rounds would be considered as positively calculated score in current study.

### Primary outcomes

Primary outcome measures of this study were calculation or not of risk scores in practice and decision of intervention in NSTE-ACS according to risk category.

### Ethical approval

This study was part of conducted clinical audit regarding management of NSTE-ACS. It was performed in accordance with the declaration of Helsinki and approved by ethical and scientific committees in Iraqi Scientific Council of Cardiology/ Iraqi Board for Medical Specializations. Informed consent to be enrolled in the study was obtained from all patients.

### Statistical analysis

Collected data were coded and input into computer using IBM SPSS Statistics version 24. Numerical variables are expressed as mean ± standard deviation, categorical variables were expressed as percentages. Statistical analysis of numerical variables was done by t-test, while that of categorical variables were done by Chi-Square test to compare frequency ratios between categories. Multiple logistic regression was used to assess predictors of invasive strategy in study population. *P* value < 0.05 is considered statistically significant [11].

## Results

One hundred fifty patients with NSTE-ACS were included in this study,108 (72%) of them were males with male to female ratio of (2.57:1). Age of patients ranges from 25 to 85 years with a mean age (59 ± 12.32) years. Tables 1 and 2 demonstrates main demographic features of patients according to GRACE and TIMI risk categories, there is the clear underuse of risk scores as TIMI score was only calculated in 8 patients (5.3%).

**Table 1:** Distribution of Initial Demographic Data According to GRACE Risk Class

Variable	GRACE Score Category			***P*** Value
	Low	Intermediate	High	
Age (Mean ± SD)	51.84 ± 9.97	60.65 ± 11.19	66.57 ± 10.94	< 0.0001
Male Gender n.(%)	45 (41.7%)	29 (26.85%)	34 (31.5%)	0.876
Female Gender n.(%)	16 (38%)	11 (26.2%)	15 (35.71%)	
Hypertension n.(%)	40 (37.4%)	30 (28.03%)	37 (34.6%)	0.434
Diabetes Mellitus n.(%)	25 (33.3%)	19 (25.3%)	31 (41.3%)	0.063
Ischemic Heart Disease n.(%)	23 (30.7%)	19 (25.3%)	33 (44%)	0.008
Smoking n.(%)	27 (55.1%)	11 (22.44%)	11 (22.44%)	0.038
Stroke n.(%)	1 (50%)	1 (50%)	0	0.571
Dyslipidemia n.(%)	12 (33.3%)	10 (27.8%)	14 (38.9%)	0.546
Family History n.(%)	30 (49.18%)	11 (18.03%)	20 (32.8%)	0.095
Prior Catheterization n.(%)	5 (29.41%)	3 (17.64%)	6 (35.3%)	0.690
Prior PCI n.(%)	12 (46.15%)	3 (11.53%)	11 (42.3%)	0.148
Prior CABG n.(%)	0	1 (33.3%)	2 (66.7%)	0.304
Chest Pain n.(%)	56 (39.2%)	38 (26.6%)	49 (34.3%)	0.19
Dyspnea n.(%)	7 (15.9%)	11 (25%)	26 (59.09%)	< 0.0001
Pulse Rate (Mean ± SD) beat per minute	76.21 ± 14.26	84.53 ± 21.41	96.67 ± 23.64	<0.0001
Baseline Systolic Blood Pressure (Mean ± SD) mmHg	138.59 ± 23.36	138.83 ± 30.56	132.14 ± 25.1	0.355
Baseline Diastolic Blood Pressure (Mean ± SD) mmHg	83.93 ± 12.27	82.7 ± 13.5	78.06 ± 12.36	0.048
Positive Troponin (%)	17 (24.6%)	17 (24.6%)	35 (50.7%)	<0.0001
GRACE Risk Score Calculation by The Treating Team (%)	0	0	0	N/A

**Table 2:** Distribution of Initial Demographic Data According to TIMI Risk Class

Variable	TIMI Score Category			***P*** Value
	Low	Intermediate	High	
Age (Mean ± SD)	53.65 ± 12.19	60.15 ± 11.34	66.76 ± 10.63	< 0.0001
Male Gender n.(%)	39 (36.11%)	52 (48.14%)	17 (15.74%)	0.798
Female Gender n.(%)	13 (30.95%)	21 (50%)	8 (19.04%)	
Hypertension n.(%)	30 (28.03%)	57 (53.27%)	20 (18.69%)	0.026
Diabetes Mellitus n.(%)	15 (20%)	43 (57.33%)	17 (22.66%)	0.001
Ischemic Heart Disease n.(%)	18 (24%)	36 (48%)	21 (28%)	0.001
Smoking n.(%)	20 (40.81%)	23 (46.93%)	6 (12.24%)	0.429
Stroke n.(%)	1 (50%)	1 (50%)	0	0.148
Dyslipidemia n.(%)	5 (13.88%)	20 (55.55%)	11 (30.55%)	0.003
Family History n.(%)	13 (21.31%)	35 (57.37%)	13 (21.31%)	0.016
Prior Catheterization n.(%)	3 (17.64%)	8 (47.05%)	6 (35.29%)	0.25
PCI n.(%)	6 (23.07%)	12 (46.15%)	8 (30.76%)	0.082
CABG n.(%)	0	1 (33.3%)	2 (66.7%)	0.055
Chest Pain n.(%)	36 (26.86%)	71 (52.98%)	36 (26.86%)	0.075
Dyspnea n.(%)	8 (18.18%)	25 (56.81%)	11 (25%)	0.016
Pulse Rate (Mean ± SD) beat per minute	79.56 ± 15.73	86.97 ± 24.92	91.24 ± 18.46	0.046
Baseline Systolic Blood Pressure (Mean ± SD) mmHg	130.08 ± 23	140.81 ± 26.69	137.56 ± 26.16	0.073
Baseline Diastolic Blood Pressure (Mean ± SD) mmHg	79.88 ± 12.65	82.52 ± 13.04	83 ± 12.58	0.452
Positive Troponin (%)	10 (14.5%)	39 (56.5%)	20 (29%)	<0.0001
TIMI Risk Score Calculation by The Treating Team (%)	1 (20%)	2 (40%)	2 (40%)	0.352

**Fig. 1:**
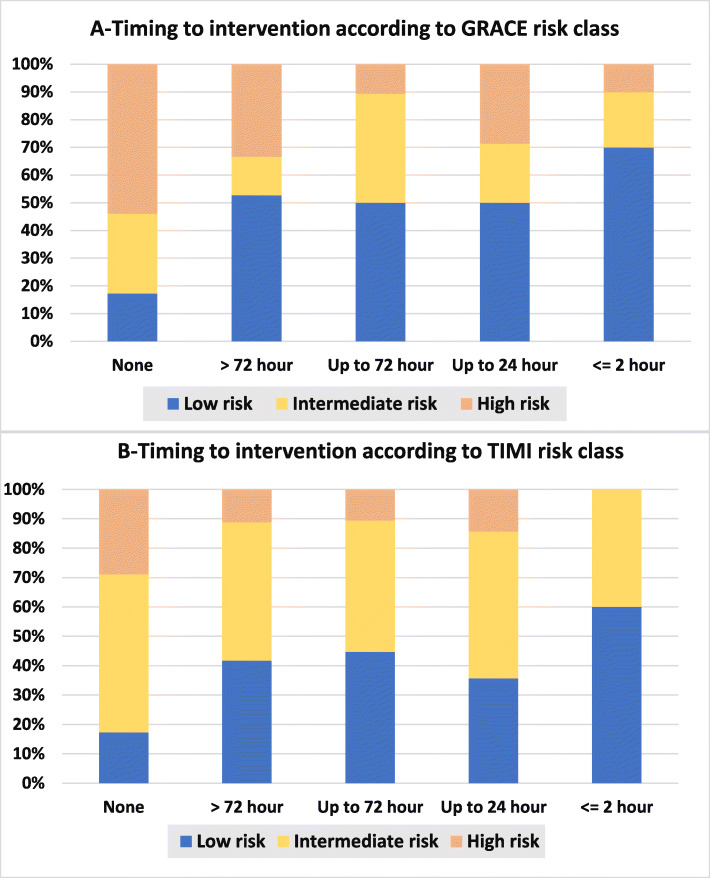
Timing to Intervention in Patients with NSTE-ACS According to GRACE and TIMI Risk Classes§. §Comparing conservative strategy versus invasive strategy according to GRACE risk class *p* < 0.0001. Comparing timing to catheterization according to GRACE risk class *p* = 0.037. Comparing conservative strategy versus invasive strategy according to TIMI risk class *p* = 0.873. Comparing timing to catheterization according to TIMI risk class *p* = 0.001

**Fig. 2:**
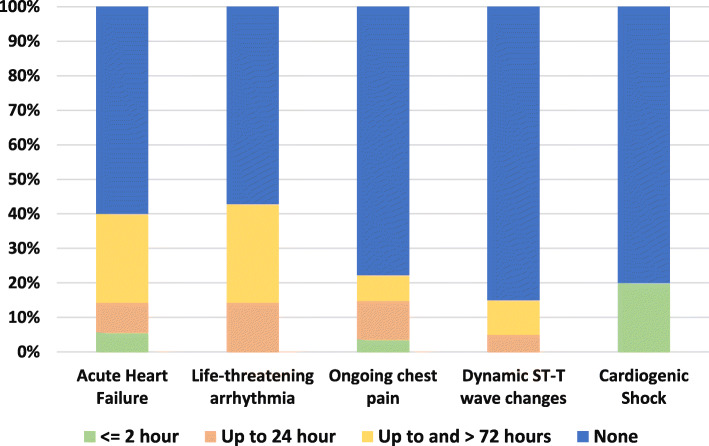
Timing to Intervention in According to Very High-Risk Criteria in Patients with NSTE-ACS

**Fig. 3:**
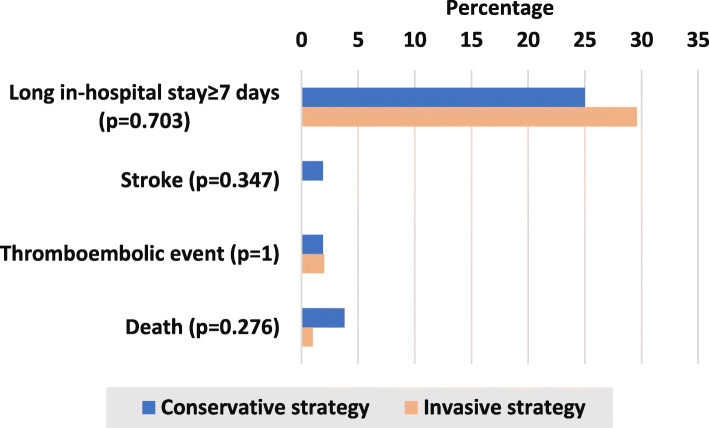
In-Hospital Outcomes of Patients with NSTE-ACS According to Management Strategy

Our results cast a light on timing to catheterization for whom managed invasively in all GRACE, TIMI risk categories and in very high-risk category as illustrated in Figs. 1 and 2. The immediate intervention in less than 2 hours was more to be used in low risk categories while the high-risk and very high-risk patients who were managed invasively were catheterized within >72 h or more frequently to be non-catheterized at all. Multiple logistic regression model was used to evaluate the impact of these independent variables on decision making as shown in Table 3. There was no significant difference in in-hospital outcomes according to management strategy as seen in Fig. 3.

**Table 3:** Multiple Logistic Regression Analysis of Variables Predicting Invasive Strategy

Variable	Standardized Coefficient Beta	Standard Error (SE)	95% CI		***P*** Value
			Lower Limit	Upper Limit	
Age	−0.189	0.003	−0.014	−0.001	0.033
Female Gender	−0.091	0.082	−0.259	0.066	0.242
GRACE Score > 140	−0.111	0.104	− 0.318	0.093	0.280
Diabetes Mellitus	−0.089	0.079	− 0.240	0.071	0.285
Prior Catheterisation	0.000	0.132	− 0.260	0.261	0.998
Prior PCI	−0.026	0.099	−0.230	0.164	0.740
Atrial Fibrillation/Flutter	−0.100	0.148	−0.477	0.111	0.22
Positve Troponin	0.148	0.079	−0.015	0.297	0.075
Acute Heart Failure	−0.142	0.101	−0.359	0.039	0.115
Ongoing Chest Pain	−0.210	0.106	−0.473	− 0.055	0.014
Cardiogenic Shock	−0.162	0.207	−0.837	− 0.019	0.04
Life-Threatening Arrhythmias	0.048	0.164	−0.224	0.425	0.541
Risk Score Calculation by The Treating Team	0.041	0.211	−0.308	0.527	0.605

## Discussion

Current study highlighted the limited use of risk scores in our daily practice in stratifying NSTE-ACS patients and illuminated the Treatment-Risk Paradox phenomenon that was remarkable in managing this patient population. This was the first designed clinical audit in cardiology field in Iraq. To date, in Iraq limited data is known about the extent of adherence of NSTE-ACS treating cardiac teams to the global guidelines regarding recommendations on risk stratification and subsequent performance of cardiac catheterization based on patients’ risk and within the recommended time frames for each risk category [12]. Moreover, this is the first study that actively and prospectively assessed use of risk scores adherence (i.e., not like prior studies that assessed use of risk scores from patients’ charts after discharge) for patients who were diagnosed as NSTE-ACS. Thus, current study can inform the policymakers to optimize use of hospital resources by the appropriate use of intervention in the right time for the right patient, many prior researchers in other countries made influential changes in practice and improved patients’ outcomes by similar studies [13–15].

The study highlights huge practice gap in form of underuse of risk scores in practice as without proper risk stratification, patient’s treatment path will certainly be misdirected, from international guidelines [5, 6, 16–18] we can conclude that risk stratification is not a (better to be done) recommendation, it is a (must be done) one. So, dropping this step will certainly lead to treatment-risk paradox.

High GRACE and TIMI risk categories as well as those with very high-risk criteria were more to be treated conservatively and even when treated invasively there was a delay in timing to catheterization compared to lower risk patients, comparative studies reported more catheterization in those with cardiogenic shock, recurrent ischemia or heart failure, and those who were managed by cardiologists rather than internists however, all our recruited patients were managed by cardiologists and cardiology fellows. Treatment-risk paradox can partly be explained by clinicians’ reluctance to perform invasive procedures in patients perceived to be at high risk for developing procedure related complications, e.g., bleeding, contrast induced kidney injury, stroke [19–21]. Our findings of such treatment-risk paradox involving even those with very-high risk criteria highlight that the entire approach to patients with clinical suspicion of NSTE-ACS should be reconsidered, regardless of the use of risk scores in clinical practice.

In a middle eastern country like Iraq, there is a vital social aspect that cannot be overlooked in real practice which is the premonition of the (Clannish Mores And Customs) and (Tribal Wergild), such premonitions push the physicians (especially interventionists) towards more conservative approach in high and very high risk patients despite the benefit from invasive strategy, since any complication or poor prognostic outcome even if scientifically justified considered unacceptable and definitely unforgivable by patients’ tribe and can cost the physician his life especially in absence of powerful system backup and law authority [22] as over the last 15 years, 20,000 doctors left Iraq seeking job abroad fearing from tribal vendettas, while 70% of Iraqi health personnel are considering leaving Iraq due to tribal customs and chaotic law authority [23]. Such socio-cultural aspects can explain not only treatment-risk paradox in current practice by impacting physicians’ decision-making but also the disparity in the rate of adherence to guidelines in form of lower adherence in these countries when compared to developed counterparts. Shedding a light on treatment decision in view of these factors with subsequent disparity can alert the policymakers to their vital role in establishing a supportive healthcare system that can help in enhancing the decision making of physicians and increasing their adherence to guidelines, there is a successful experience in this regard in Iraq [22].

Adherence to guidelines in term of decision and timing of intervention in NSTE-ACS play a key role in improving patients’ outcomes, as the highest benefit was reported in high risk patients who were treated invasively in form of absolute risk reduction in addition to decrease recurrent ischemia, subsequent rehospitalization and revascularization as well as cardiovascular death and all-cause mortality at 30-days, 12 months and 5-years follow-up [15, 24]. Recent Meta-analysis [25] provides strong evidence regarding the highest benefit of early invasive strategy in higher risk NSTE-ACS in term of lower major adverse cardiac events and recurrent ischemic events in parallel to what was reported by VERDICT trial [26]. However, we did not observe significant differences in in-hospital outcomes according to use of invasive strategy and this can be explained by the delayed catheterization if done in high-risk patients which can partially waive its benefit or catheterizing low risk-patients who usually benefit less from intervention compared to the higher risk categories.

Not only for improving patients’ outcomes but being an emerging country with limited resources, absent health insurance policy and absent non-governmental funding with a few numbers of cardiac centres, in addition to shortage of staff and above all; shortage of materials and supplies for intervention, sometimes with total absence for remarkable periods. All that necessitate that our resources and efforts should be targeted towards the higher risk class to optimize patients’ outcomes.

To overcome treatment-risk paradox in practice we recommend specifying a highlighted box for documenting the risk of patients in hospital records once admitted to CCU to motivate the residents to consider risk stratification in practice. We suggest using teaching posters and pocket guides to promote adherence to guidelines. We recommend that mentors and trainers emphasize more on proper risk stratification and adherence to evidence-based management during grand rounds and meetings. In high volume centres, we propose prioritizing the higher risk patients and emergency procedures over elective ones in resource-limited settings. Moreover, it is crucial to conduct clinical audits with further communication with decision-makers to ensure proper feedback to conduct quality improvement projects to optimize patients’ outcomes and achieve cost-effectiveness of performed measures and procedures.

### Study limitations

Larger sample size is warranted in future to further validate the results, the strict selection of cases especially regarding the mandatory presence of the investigator during daily follow-up limited substantially the sample size in addition to absent electronic database and lack of research collaborators. Long-term prognostic outcomes resulting from the treatment-risk paradox can add more insights to the impact of this practice gap to catch the attention of healthcare providers to the importance of adherence to guidelines in daily practice.

## Conclusions

There is striking underuse of risk scores in practice that can contribute to treatment-risk paradox in managing NSTE-ACS in form of depriving those with higher risk from invasive strategy despite being the most beneficiaries. The paradox did not only involve the very high-risk patients but also the very high-risk criteria like ongoing chest pain and cardiogenic shock predicted conservative approach, this highlights that the entire approach to patients with NSTE-ACS should be reconsidered, regardless of the use of risk scores in clinical practice. Audit programs activation in middle eastern countries can inform policymakers to put a limit to the treatment-risk paradox for better cardiovascular care and outcomes.

## Data Availability

The data sets used and/or analysed during the study are available from the corresponding author on reasonable request.

## Abbreviations

ACS: Acute Coronary Syndrome
CABG: Coronary Artery Bypass Graft
CAD: Coronary Artery Disease
CCU: Coronary Care Unit
ECG: Electrocardiogram
GRACE: Global Registry of Acute Coronary Events
LBBB: Left Bundle Branch Block
NSTE-ACS: Non-ST Elevation Acute Coronary Syndromes
NSTEMI: Non ST Elevation Myocardial Infarction
PCI: Percutaneous Coronary Intervention
STEMI: ST Elevation Myocardial Infarction
TIMI: Thrombolysis in Myocardial Infarction
